# High retention among key populations initiated on HIV pre‐exposure prophylaxis in Kigali City, Rwanda

**DOI:** 10.1002/jia2.26392

**Published:** 2024-11-20

**Authors:** Athanase Munyaneza, Kiran Bhutada, Qiuhu Shi, Natalia Zotova, Etienne Nsereko, Benjamin Muhoza, Gallican Kubwimana, Gad Murenzi, Laetitia Nyirazinyoye, Kathryn Anastos, Viraj V. Patel, Jonathan Ross

**Affiliations:** ^1^ Research for Development (RD Rwanda) Kigali Rwanda; ^2^ University of Rwanda School of Public Health Kigali Rwanda; ^3^ Albert Einstein College of Medicine New York New York USA; ^4^ New York Medical College Valhalla New York New York USA

**Keywords:** PrEP retention, key populations, female sex workers, men who have sex with men, Kigali, Rwanda

## Abstract

**Introduction:**

Key populations (KPs) including female sex workers (FSWs) and men who have sex with men (MSM) in sub‐Saharan Africa are disproportionately impacted by HIV. Despite the increasing availability of pre‐exposure prophylaxis (PrEP), data on retention remain limited. This study assessed PrEP retention at 1 and 12 months among Rwandan FSWs and MSM.

**Methods:**

We analysed routine clinical data on adult FSWs and MSM receiving PrEP care from 11 health facilities in Kigali, Rwanda between 2019 and 2022. Retention was defined as attendance at regularly scheduled appointments for a PrEP refill. We used logistic regression to assess associations between demographic and clinical characteristics and retention at 1 and 12 months.

**Results:**

Among 2043 PrEP initiators, 1343 (66%) were FSWs and 700 (34%) were MSM. FSWs reported a median number of eight sexual partners in the prior 7 days, 70% reported condomless sex and 94% considered themselves at high HIV risk. About 1239 (92%) and 1032 (77%) were retained at 1 and 12 months, respectively. One‐month retention was lower among FSWs living with others (OR 0.59, 95% CI: 0.35−0.99; ref: living alone) or with low HIV risk perception (OR 0.12, 95% CI: 0.04−0.29). At 12 months, low HIV risk perception remained statistically significant (aOR 0.20, 95% CI: 0.12−0.32). At PrEP initiation, MSM reported a median of four sexual partners in the prior 12 months, 88% reported condomless sex and 72% considered themselves at high HIV risk. Retention rates were 96% at 1 month and 82% at 12 months. At 1 month, retention was higher among MSM with some education (OR 12.74, 95% CI: 2.74−70.93; ref: no education). At 12 months, retention was lower among MSM with part‐time employment (aOR 0.29, 95% CI: 0.11, 0.76), students (aOR 0.12, 95% CI: 0.04, 0.37) and unemployed (aOR 0.12, 95% CI: 0.05, 0.28); ref: full‐employed) and those unaware of PrEP at baseline (aOR 0.15, 95% CI: 0.10, 0.23).

**Conclusions:**

We observed very high rates of PrEP retention among Rwandan FSWs and MSM. Predictors of retention included living situation, employment status, HIV risk perception and low PrEP awareness, but differed between FSWs and MSM. These findings suggest that targeted awareness campaigns tailored to different KPs could improve PrEP retention in care.

## 1 INTRODUCTION

HIV disproportionately impacts key populations (KPs)—female sex workers (FSWs), men who have sex with men (MSM), injection drug users and others—including in sub‐Saharan Africa (SSA), which remains the global region most impacted by HIV. In 2021, KPs and their sexual partners accounted for 51% of new HIV acquisitions in this region [1]: FSWs comprised 15%, MSM accounted for 6%, clients of sex workers and sex partners of KP made up 26% and the remaining 4% belonged to other categories [1]. The increased vulnerability among KPs is primarily due to their engagement in high‐risk sexual activities [2] and the stigma and discrimination to which they are subjected [3]. Within KP environments, these social challenges act as substantial barriers, rendering it more difficult for them to acquire information on accessible HIV prevention services and to utilize these services effectively.

Pre‐exposure prophylaxis (PrEP) can substantially decrease new HIV acquisition, with consistent adherence reducing the risk of HIV acquisition by nearly 99% [4]. Despite a high willingness among KPs in SSA to use PrEP [5, 6, 7], multiple barriers impact access and adherence for these populations, including competing economic and social needs, and limited access to healthcare, education and employment [8, 9]. These structural barriers are compounded by multiple stigmas faced by KPs related to sexual identity, sex work and HIV [10]. At the health‐facility level, while trust in certain healthcare providers can positively impact PrEP retention for KPs [11], persistent barriers exist due to healthcare providers’ limited awareness of PrEP [12] and many health services, both private and public, that are not specifically targeted at KPs are often unfriendly and discriminatory, leading to avoidance by KPs.

Rwanda has made significant progress in expanding PrEP access, aligning with its commitment to reduce new HIV acquisition and enhance the health of KPs. The country's prevention, treatment and care guidelines emphasize the key role of PrEP in HIV prevention [13]. The limited data available to date indicate relatively high retention among KPs initiating PrEP in Rwanda, with estimates from 53% to 82% [14, 15] after 1 year of follow‐up. However, those studies revealed significant gaps or missing information and involved an exceptionally small MSM sample size. To bridge this gap, we conducted a study to assess retention on PrEP and factors predicting this outcome.

## 2 METHODS

### 2.1 Study design, setting and population

This was a retrospective cohort study analysing routine clinical data from FSWs and MSM evaluated for PrEP at 11 (31%) primary‐care health facilities in the City of Kigali, Rwanda's capital, between 2019 and 2022. Rwanda, with a population of around 13 million [16], has an HIV prevalence of 3% among adults [17], but it is significantly higher among FSWs (up to 57%) [2] and MSM (11%) [18]. Annual HIV incidence in adults is 0.08% [17], compared to 1.36% among FSWs [19]. No studies have reported HIV incidence among Rwanda MSM. Rwandan guidelines prioritize KPs, including FSWs and MSM, for HIV control efforts such as PrEP [20]. Recent studies have estimated the population of FSWs between 8328 and 22,806 [21] and that of MSM at approximately 18,100 individuals [22], with the biggest proportion (approximately 8000 MSM) located in Kigali [22].

In Rwanda, PrEP is recommended for HIV‐negative individuals at high risk of HIV acquisition and is available in most health centres as oral tenofovir disoproxil fumarate‐emtricitabine [20]. Eligibility requires individuals to be 18 or older, have a negative HIV test, normal kidney function and a commitment to daily medication. Patients undergo initial clinical and lab evaluations, prior to initiation, follow‐up visits to assess adherence (through self‐report and pill counts), assess symptoms of sexually transmitted infections (STIs), conduct HIV testing and dispense PrEP if indicated occur at 1 month and every 3 months thereafter, with annual kidney function checks [20].

The study sourced data from 11 facilities in Kigali, including eight participating in the Central Africa International epidemiologic Databases to Evaluate AIDS (CA‐IeDEA) and three clinics recognized by KP community members and non‐government organizations for providing KP‐friendly services. Rwanda's PrEP implementation began in 2019, with over 29,000 individuals having initiated PrEP as of November 2023 [23].

### 2.2 Data source

At primary healthcare facilities screening, initiation and follow‐up visit data for KPs on PrEP are recorded in paper medical files that include: data on eligibility for PrEP, awareness of and willingness to initiate PrEP, sexual health information such as number and type of sexual partners, recent STI symptoms, and results of serological testing for HIV and renal function. For this study, data were extracted from paper medical files into a Research Electronic Data Capture (REDcap) database by data entry personnel at the health facilities, interns or study staff. To ensure quality assurance, weekly calls were scheduled between principal investigators and coordinators to discuss the data extraction and entry process, and to review the entered data.

### 2.3 Study variables and measurements

Our primary objective of the study was to assess PrEP retention—defined as attendance at scheduled appointments—at 1 and 12 months after initiation. We also examined the proportion of participants who were aware of PrEP prior to the initial visit (defined as: “*Before today, have you ever heard of HIV‐uninfected people taking ARV every day to reduce the risk of getting HIV*”), willing to use PrEP at baseline (defined as: “*willing to take ARV every day to lower the chances of getting HIV*”).

Additional variables included: (1) socio‐demographic information, such as the KP category (FSW, MSM, other), living situation (alone vs. with others including living with parents/family members, cohabiting, live with roommates), education level (none vs. some) and employment status (full‐time employment, part‐time employment, student or unemployed); (2) sexual history and HIV risk perception data, including the number of sexual partners within the last 7 days for FSWs and 12 months for MSM preceding PrEP screening, condomless sex prior to initial visit (yes or no) and self‐perceived high risk of HIV acquisition (yes or no); and (3) information on STI symptoms in the preceding 12 months, including whether individuals had ever been screened for or diagnosed with an STI during that time.

### 2.4 Data analysis

Bivariate logistic regression taking into account the number of individuals who initiated PrEP with complete data was used to examine the associations between baseline characteristic variables and retention at month 1 and at month 12, reported as odds ratios (ORs) with 95% confidence intervals (CIs). Multivariate models included baseline characteristics that were associated with outcome variables at an alpha of <0.05, had sufficient available data or were felt to be clinically relevant based on prior literature. Associations were reported at an alpha level of 0.05 as adjusted odds ratios (aORs) with 95% CIs for PrEP retention at 1 and 12 months. To assess the impact of missingness on model results, we compared the characteristics of individuals included in models and those not included because of missing data for some variables (Table S1: Comparison of individuals included vs. excluded from multivariable models). In a sensitivity analysis, we examined retention on PrEP using a repeated measures approach, utilizing generalized estimating equations (GEE) to account for within‐individual correlation between time points.

Finally, we used descriptive statistics to examine associations between health facility (friendly vs. mainstream health centre, larger vs. smaller PrEP programme) and retention at 1 and 12 months.

### 2.5 Ethical consideration

This study was approved by the Rwanda National Ethics Committee (700/RNEC/2021) and by the Institutional Review Board (IRB) of the Albert Einstein College of Medicine (IRB number: 2020–12619). Both committees waived a requirement for informed consent given the second data use of routine clinical data for this study. We assigned study identification numbers to replace identifying information for all patients whose data we extracted into the study database.

## 3 RESULTS

### 3.1 FSW and MSM characteristics

Data from 2043 individuals who initiated PrEP were included in the analysis (Table 1), of whom 1343 were FSWs and 700 were MSM. At baseline, 71% of FSWs reported that they lived alone, 75% had attained some form of education (primary or higher) and 51% were unemployed. FSWs at baseline reported a median of eight sexual partners in the prior 7 days and, the majority (69%) reported condomless sex. Nearly, all (92%) considered themselves at high HIV risk, and 21% reported ever being diagnosed or treated for STIs. FSWs with lower HIV risk perception had more sexual partners (13 vs. 8, *p*<0.001) and higher STI rates (37% vs. 24%, *p*<0.05) compared to those with high‐risk perception. Awareness and willingness to use PrEP were high at 62% and 93%, respectively.

**Table 1:** Characteristics, awareness of, willingness to use PrEP among study participants

	KP category (*n* = 2043)	
	FSW (1343)	MSM (700)
KP characteristics	Frequency (%), Median	Frequency (%), Median
**Living situation**		
Live alone	960 (71.48)	345 (49.29)
Live with others	174 (12.95)	334 (47.71)
Missing	209 (15.57)	21 (3)
**Education level**		
None	129 (9.60)	14 (2)
Some education (primary or higher)	1012 (75.35)	680 (97.14)
Missing	202 (15.05)	6 (0.86)
**Employment status**		
Full‐time	62 (4.61)	98 (14)
Part‐time	357 (26.58)	255 (36.42)
Student	21 (1.56)	55 (7.85)
Unemployed	695 (51.48)	272 (2.85)
Missing	208 (15.48)	20 (2.85)
**Median number of sexual partners (in the last 12 months)**	8.0 (3.5, 15.5)	4.0 (2, 6)
**Condomless sex prior to initial visit**		
Yes	923 (68.73)	542 (77.43)
No	402 (29.93)	67 (9.57)
Missing	18 (1.34)	91 (13)
**Consider oneself to be at high risk of HIV**		
Yes	1236 (92.03)	439 (62.71)
No	81 (6.03)	175 (25)
Missing	26 (1.94)	86 (12.29)
**Aware of PrEP prior to initial visit**		
Yes	830 (61.80)	495 (70.71)
No	481 (35.82)	119 (17)
Missing	32 (2.38)	86 (12.29)
**Willing to use daily PrEP**		
Yes	1248 (92.93)	596 (85.14)
No	59 (4.39)	15 (2.14)
Missing	36 (2.68)	89 (12.71)
**Self‐reported diagnosis and treatment for STI in prior 12 months**		
Yes	287 (21.37)	307 (43.86)
No	849 (63.22)	382 (54.57)
Missing	207 (15.41)	11 (1.57)

Abbreviations: KP, key population; PrEP, pre‐exposure prophylaxis; STI, sexually transmitted infection.

Among MSM, 49% lived alone, 97% had completed some education and 36% were unemployed. They reported a median of four sexual partners in the past year, with 77% practicing condomless sex. Sixty‐three percent perceived themselves at high risk for HIV, and 44% had been diagnosed or treated for STIs. MSM with lower risk perception had fewer sexual partners (2 vs. 4, *p*<0.001), but no significant differences in STI history. Awareness and willingness to use PrEP were also high at 71% and 85%, respectively. A sensitivity analysis (Table S1: Comparison of individuals included vs. excluded from multivariable models) revealed no significant differences in population characteristics with complete data.

### 3.2 PrEP retention and associated factors

Overall retention in the study population was high, with 1913 (94%) returning at 1 month after initiation and 1605 (79%) retained in care at 12 months (Table S2: PrEP appointment retention among individuals at 11 Kigali health centres, 2019–2022). Relatively, few patients who remained in care missed appointments (Figure S1. Retention patterns among PrEP patients at 11 health centres in Kigali). Among FSWs, PrEP retention was 92%, 90%, 86%, 78% and 76% at 1, 3, 6, 9 and 12 months after initiation, respectively. In bivariate analysis (Table 2), FSWs who did not perceive themselves to be at high risk of HIV acquisition (OR: 0.13, 95% CI: 0.15–0.31) were less likely to be retained at 1 month compared to those who perceived themselves to be at high risk. In the multivariable analysis, 1‐month retention was lower among FSWs who lived with others (vs. alone: aOR: 0.59, 95% CI: 0.35−0.99) or with low HIV risk perception (vs. high: aOR: 0.11, 95% CI: 0.04−0.29). FSWs with part‐time employment (aOR: 2.36; 95% CI: 1.28, 4.32) and students (aOR: 5.51; 95% CI: 1.00−30.24) had significantly higher odds of PrEP retention at 1 month compared to those with full‐time jobs.

**Table 2:** Factors associated with PrEP retention at 1‐ and 12‐month appointments among FSWs who initiated PrEP with complete data

	FSW					
	1‐month PrEP retention			12 months PrEP retention		
KP characteristics	Return 1 month	OR (95% CI)	aOR (95% CI)	Return 12 months	OR (95% CI)	aOR (95% CI)
**Living situation**						
Live alone	849	**Ref**		750	**Ref**	
Live with others	147	0.62 (0.35, 1.07)	**0.59 (0.35, 0.99)**	134	0.94 (0.61, 1.44)	0.96 (0.56, 1.65)
**Education level**						
None	112	**Ref**		95	**Ref**	
Some education (primary or higher)	884	1.22 (0.63, 2.38)	1.19 (0.81, 1.75)	789	1.40 (0.89, 2.21)	1.38 (0.79, 2.39)
**Employment status**						
Full‐time	52	**Ref**		50	**Ref**	
Part‐time	323	2.42 (0.96, 6.07)	**2** **.36 (1.28, 4.32)**	286	0.94 (0.44, 2.01)	0.88 (0.50, 1.56)
Student	21	2.83 (0.33, 24.41)	**5.51 (1.00,30.24)**	16	0.48 (0.15, 1.56)	0.54 (0.16, 1.80)
Unemployed	600	1.44 (0.63, 3.33)	1.40 (0.68, 2.90)	532	0.77 (0.37, 1.61)	0.74 (0.50, 1.11)
**Condomless sex since prior to initial visit**						
Yes	723	**Ref**		637	**Ref**	
No	273	0.69 (0.43, 1.11)	0.99 (0.52, 1.88)	247	0.98 (0.69, 1.38)	1.11 (0.65, 1.88)
**Consider oneself to be at high risk of HIV**						
Yes	953	**Ref**		842	**Ref**	
No	43	**0.13 (0.07, 0.23)**	**0.11 (0.04, 0.29)**	42	**0.39 (0.23, 0.67)**	**0.35 (0.12, 0.96)**
**Aware of PrEP prior to initial visit**						
Yes	623	**Ref**		548	**Ref**	
No	373	0.85 (0.53, 1.34)	1.30 (0.80, 2.11)	336	1.06 (0.77, 1.47)	1.22 (0.57, 2.60)
**Self‐reported diagnosis and treatment for STI in prior 12 months**						
Yes	252	**Ref**		225	**Ref**	
No	744	0.95 (0.56, 1.61)	0.84 (0.42, 1.65)	659	0.94 (0.65, 1.34)	0.90 (0.51, 1.57)

*Note*: The bold numbers indicate only the statistically significant results.

Abbreviations: FSW, female sex worker; PrEP, pre‐exposure prophylaxis; STI, sexually transmitted infection.

At 12 months, in the bivariate analysis, FSWs who did not perceive themselves to be at high risk of HIV acquisition (OR: 0.39, 95% CI: 0.23–0.67) were less likely to be retained to PrEP compared to those who perceived themselves to be at high risk. In the multivariable analysis, FSWs who did not perceive a higher risk of HIV acquisition (aOR: 0.35, 95% CI: 0.12–0.96) were also less likely to be retained. A repeated measures sensitivity analysis using GEE showed similar results (Table S3: Repeated measures analysis of PrEP retention in FSW using GEE across 12‐month visits).

Among MSM, retention was 96%, 95%, 86%, 82% and 82% at 1, 3, 6, 9 and 12 months, respectively. At 1 month, bivariate analysis (Table 3) showed that MSM with some form of education were more likely to be retained on PrEP compared to those with no education (OR: 9.89, 95% CI: 1.94–50.56). In multivariable analysis, MSM with some form of education (vs. no education: aOR: 12.89, 95% CI: 2.29.3–70.93) were significantly more likely to be retained.

**Table 3:** Factors associated with PrEP retention at 1‐ and 12‐month appointments among MSM who initiated PrEP with complete data

	MSM					
	1‐month PrEP retention			12 months PrEP retention		
KP characteristics	Return 1 month	OR (95% CI)	aOR (95% CI)	Return 12 months	OR (95% CI)	aOR (95% CI)
**Living situation**						
Live alone	303	**Ref**		280	**Ref**	
Live with others	251	2.76 (0.75, 10.14)	3.08 (0.55, 17.09)	225	0.91 (0.54, 1.55)	**0.73 (0.55, 0.96)**
**Education level**						
None	10	**Ref**		12	**Ref**	
Some education (primary or higher)	544	**9.89 (1.94, 50.56)**	**12.74 (2.29, 70.93)**	493	Fail to converge	
**Employment status**						
Full‐time	83	**Ref**		80	**Ref**	
Part‐time	208	0.84 (0.09, 8.15)	0.74 (0.10, 5.52)	201	1.00 (0.31, 3.30)	0.53 (0.19, 1.46)
Student	36	0.22 (0.02, 2.47)	0.12 (0.01, 2.62)	33	0.33 (0.08, 1.31)	**0.25 (0.07, 0.83)**
Unemployed	227	0.39 (0.05, 3.22)	0.45 (0.18, 1.12)	191	**0.22 (0.08, 0.64)**	**0.20 (0.04, 0.92)**
**Condomless sex prior to initial visit**						
Yes	488	**Ref**		442	**Ref**	
No	66	1.62 (0.21, 12.68)	2.46 (0.36, 16.68)	63	2.07 (0.73, 5.89)	1.58 (0.82, 3.07)
**Aware of PrEP prior to initial visit**						
Yes	446	**Ref**		433	**Ref**	
No	108	0.54 (0.16, 1.80)	0.68 (0.11, 4.35)	72	**0.09 (0.05, 0.16)**	**0.15 (0.03, 0.70)**
**Self‐reported diagnosis and treatment for STI in prior 12 months**						
Yes	212	**Ref**		188	**Ref**	
No	342	0.72 (0.22, 2.36)	0.66 (0.17, 2.54)	317	1.39 (0.82, 2.36)	1.53 (0.58, 4.09)

*Note*: The bold numbers indicate only the statistically significant results.

Abbreviations: MSM, men who have sex with men; PrEP, pre‐exposure prophylaxis; STI, sexually transmitted infection.

At 12 months, in the bivariate analysis, MSM were less likely to be retained if they were unemployed (OR: 0.22, 95% CI: 0.08−0.64; ref: employed full‐time) or not aware of PrEP at baseline (OR: 0.09, 95% CI: 0.05−0.16; ref: aware of PrEP). In multivariable analysis, 12‐month retention was lower among MSM living with others (vs. alone: aOR: 0.73, 95% CI: 0.55−0.96), those who were students (aOR: 0.25, 95% CI: 0.07−0.83) or unemployed (aOR: 0.20, 95% CI: 0.04−0.92) compared to those with full‐time employment, and among those not aware of PrEP at baseline (aOR: 0.15, 95% CI: 0.03−0.70; ref: aware of PrEP). The repeated measures sensitivity analysis using GEE showed similar results (Table S4: Repeated measures analysis of PrEP retention in MSM using GEE across 12‐month visits).

In descriptive analyses examining facility‐level characteristics, we observed higher retention at both 1 and 12 months at friendly compared to mainstream health centres (1 month: 99% vs. 90%; 12 months: 90% vs. 71%, *p*<0.001 for both) and at larger PrEP programmes compared to smaller (1 month: 96% vs. 91%; 12 months: 88% vs. 65%, *p*<0.001 for both) (Table S2: PrEP appointment retention among individuals at 11 Kigali health centres, 2019–2022).

## 4 DISCUSSION

In this study of routine clinical data from a convenience sample of 11 health centres in Kigali, Rwanda, we observed very high PrEP retention rates among both FSWs and MSM, at both 1‐ and 12‐month time points. Retention in PrEP care was less likely among patients living with others compared to those living alone, patients reporting a lack of PrEP awareness prior to initiation compared to those already aware and patients with low compared to high HIV risk perception. While routine clinical data limited deeper insights, our findings suggest that living arrangements affect privacy and support, impacting PrEP retention. Targeted interventions to address HIV risk perception and enhance PrEP awareness for KPs in Rwanda are crucial.

The high rate of retention we observed is similar to findings from some studies in SSA, though markedly higher than others. A systematic review of FSWs in SSA including data from eight studies reported a pooled PrEP retention of 76% at 6 months [24], while studies of FSWs and MSM conducted in Rwanda and Senegal demonstrated 12‐month retention rates of 73–88% [15, 25]. However, other investigators have reported substantially lower PrEP retention in other SSA settings such as Kenya, Benin, Swaziland and South Africa, ranging from 23% to 59% [26, 27, 28, 29]. The high rate of retention in our study may be due to several factors. First, our prior research demonstrated high PrEP awareness and willingness to use PrEP among KPs in Rwanda [30]; other settings with lower awareness and education may struggle with lower retention. The observed results may also be a function of social norms related to HIV stigma and PrEP as well as the general level of PrEP awareness and availability of PrEP services; in our qualitative work, we highlighted a strong positive role that social networks play in contributing to PrEP uptake and retention among Rwandan FSWs and MSM [31].

Similarly, a study in Uganda showed that FSWs viewed health facilities as welcoming, which positively influenced their engagement with PrEP services [32]. Individuals who chose to visit these clinics for PrEP often remained committed due to the supportive environment. Additionally, local contextual factors may have positively impacted retention, including community mobilizers who disseminate PrEP information and actively encourage eligible individuals to visit clinics, as well as economic transportation incentives that were provided in many health centres as part of PEPFAR‐related services [33].

Of note, a study of Rwandan FSWs conducted by Mubezi et al. (including data from two health centres included in the present study) reported a 12‐month retention on PrEP of 53% among FSWs; substantially lower than our findings. The high retention observed in our study may be due to differences in study design (e.g. cross‐sectional vs. cohort), duration of follow‐up time, and in particular data source. We actively extracted data from individual paper medical files at each health centre and entered them into a study database, helping to minimize missing data including follow‐up appointment attendance, while Mubezi et al. utilized a PEPFAR database that may have been less comprehensive.

Our results indicate that retention in PrEP care varies between FSWs and MSM, with different factors affecting each group. FSWs living with others showed lower retention, possibly due to privacy or support issues, while low HIV risk perception was linked to reduced retention, particularly among those with more sexual partners and STI histories. For MSM, higher education improved retention, reflecting a better understanding of PrEP. Employment status also played a role, with part‐time work and student status linked to better retention for FSWs. These findings emphasize the need for tailored interventions addressing privacy, risk perception and education to improve PrEP retention.

At 12‐month appointments, we identified distinct factors affecting PrEP retention among FSWs and MSM, including living situations, employment status, HIV risk perception and baseline awareness of PrEP. For MSM, those living with others had lower odds of retention, indicating that less private or supportive environments might hinder their engagement in care, aligning with previous qualitative findings [31] as well as other studies describing lower retention among MSM with unmet social determinants of health and basic needs challenges [34]. Conversely, FSWs did not show significant retention differences based on the living situation; however, part‐time employment or student status correlated with higher retention, possibly due to reduced job stress and more flexible schedules. Notably, we were not able to assess whether FSWs considered sex work as formal employment. Conversely, MSM who were students or unemployed had lower retention odds compared to those employed full‐time, suggesting that better economic circumstances and access to healthcare resources support retention. FSWs with low perceived HIV risk demonstrated significantly lower retention, despite higher‐risk behaviours, raising concerns about awareness and engagement in care. Other studies have also reported low perception of HIV risk to be associated with low retention among PrEP users [35]. Interestingly, FSWs who were unaware of PrEP prior to their first visit showed higher retention rates, while MSM without prior awareness had significantly lower odds of retention. This disparity may reflect differences in social networks, information sources and motivation between FSWs and MSM. Further research is essential to explore how these factors impact engagement in PrEP care and to develop targeted interventions for both populations.

Although PrEP awareness prior to initiation was associated with retention, overall, we observed high levels of awareness of and very high levels of willingness to use PrEP among Rwandan FSWs and MSM. Our results are similar to studies of KPs conducted elsewhere in SSA, including Tanzania, Uganda, Nigeria and Kenya [27, 36, 37, 38], and two systematic reviews [39, 40] reporting robust awareness of PrEP, and very high levels of willingness to use it among both FSWs and MSM. Prior work we have conducted in Rwanda suggests that among MSM, awareness of PrEP has increased over time, and willingness to use it remains very high [5, 30]. Given this, policy efforts should focus on leveraging this positive attitude to improve PrEP accessibility and uptake among FSWs and MSM. Implementing targeted educational campaigns, ensuring transparent access to PrEP services and promotion of, community‐led monitoring of the programme would leverage this favourable disposition, contributing to a more holistic and effective HIV prevention strategy for these KPs.

We observed higher retention at friendly health centres compared to mainstream centres, and at larger compared to smaller PrEP programmes. Notably, the large majority of MSM, whose retention was higher than FSWs, were seen in these health centres. While we are not able to definitively determine which underlying factors contributed to higher retention in these clinics, these findings suggest that programmes with specific training in caring for KPs and with more experience providing PrEP may have better outcomes; additional research in this area is needed.

## 5 LIMITATIONS

This study has several limitations due to the reliance on routinely collected clinical data, which may lack information on other factors influencing PrEP use, such as age or specific health behaviours, due to documentation issues or missing data. While we aimed to include all FSWs and MSM initiating PrEP at the selected health facilities, some data may still be absent, potentially affecting the overall representativeness of our findings. The study focused on a convenience sample from urban health centres, including three designated “friendly” facilities, which may not reflect the experiences of all FSWs and MSM in Rwanda, particularly in rural areas.

Additionally, Rwanda's unique care delivery strategies, such as community mobilizers promoting PrEP within their communities, may have enhanced individual awareness and influenced engagement levels during screening. This proactive outreach could skew results when compared to areas lacking similar programmes. Furthermore, the accuracy of self‐reported data on the number of sexual partners among MSM could introduce recall bias due to the 12‐month reporting period. We also faced challenges with inconsistent recording of PrEP adherence in patient files, limiting our ability to measure effective use among FSWs and MSM accurately. Previous research has indicated that retention does not always correlate with actual adherence [41], emphasizing the need for comprehensive measurement methods. No positive HIV test results were observed in the study data, likely because seroconversions occurred among patients lost to follow‐up or whose clinical records were transferred to HIV programmes; we, therefore, do not report on seroconversion, an important measure of PrEP effectiveness. To address these limitations, future research should expand data collection efforts across all healthcare facilities offering PrEP, incorporating both quantitative and qualitative approaches. This broader strategy could yield deeper insights into factors affecting the PrEP cascade and inform future interventions.

## 6 CONCLUSIONS

This study highlights high levels of PrEP retention among both FSWs and MSM in Rwanda. Our findings point to the successful implementation of PrEP in Rwanda and suggest the potential of PrEP to become a widely adopted HIV prevention strategy for KPs in SSA and beyond. Though the majority of patients in this study remained in care, retention was less likely among those with low HIV risk perception and low PrEP awareness prior to initiation. Campaigns to sensitize KPs about HIV risk and PrEP should be considered as strategies to promote awareness, improve PrEP engagement and retention, and ultimately help reduce the impact of HIV.

## COMPETING INTERESTS

The authors declare no competing interests.

## AUTHORS’ CONTRIBUTIONS

AM, VVP and JR were key contributors to the study protocol development. AM oversaw data extraction, while AM, QS, KB, NZ and JR performed data analysis. AM led the manuscript drafting, with significant contributions from KB, QS, NZ, EN, GM, LN, JR, VVP and KA on design and editing. BM, GK and JR were involved in drafting and revising the work. All authors approved the final manuscript version.

## FUNDING

This study was funded by the Central Africa IeDEA which is also supported by the National Institutes of Health's National Institute of Allergy and Infectious Diseases (NIAID), the Eunice Kennedy Shriver National Institute of Child Health & Human Development (NICHD), the National Cancer Institute (NCI), the National Institute on Drug Abuse (NIDA), the National Heart, Lung, and Blood Institute (NHLBI), the National Institute on Alcohol Abuse and Alcoholism (NIAAA), the National Institute of Diabetes and Digestive and Kidney Diseases (NIDDK), the Fogarty International Center (FIC), the National Library of Medicine (NLM) and the Office of the Director (OD) under Award Number U01AI096299 (Central Africa‐IeDEA).

## Supporting information




**Figure S1**: Retention patterns among PrEP patients at 11 health centers in Kigali (N = 2043)

**Table S1**: Comparisons between individuals included in multivariable models and individuals excluded from models.
**Table S2**: Retention at scheduled PrEP appointments among 2043 individuals initiating PrEP at 11 health centers in Kigali, Rwanda, 2019 2022.
**Table S3**: Repeated measures analysis of PrEP retention among female sex workers utilizing generalized estimating equations, including data from 1‐, 3‐, 6‐, 9‐ and 12‐month visits (N = 1053).
**Table S4**: Repeated measures analysis of PrEP retention among men who have sex with men utilizing generalized estimating equations, including data from 1‐, 3‐, 6‐, 9‐ and 12‐month visits (N = 558).
**Table S5**: One‐ and twelve‐month PrEP retention at 11 health centers in Kigali, Rwanda.

## Data Availability

The data that support the findings of this study are available on request from the corresponding author or senior author.
