# Supplementary material for: High retention among key populations initiated on HIV pre‐exposure prophylaxis in Kigali City, Rwanda
Source: J Int AIDS Soc. 2024 Nov 20;27(11):e26392. doi: 10.1002/jia2.26392 (PMC11578929; doi:10.1002/jia2.26392)
Supplement: Supplementary file 1 — Figure S1: Retention patterns among PrEP patients at 11 health centers in Kigali (N = 2043) [file JIA2-27-e26392-s001.docx]

**Supplementary Figure 1.** Retention patterns among PrEP patients at 11 health centers in Kigali (N=2043)

| **Month of follow-up** | | | | | | **Relative frequency of retention pattern** | **Percent** | | **Cumulative percent** |
| --- | --- | --- | --- | --- | --- | --- | --- | --- | --- |
| **1** | | **3** | **6** | **9** | **12** |  |  |  |  |
|  | |  |  |  |  | 1449 | 70.9 | | 70.9 |
|  | |  |  |  |  | 120 | 5.9 | | 76.8 |
|  | |  |  |  |  | 102 | 5.0 | | 81.8 |
|  | |  |  |  |  | 99 | 4.9 | | 86.7 |
|  | |  |  |  |  | 71 | 3.5 | | 90.2 |
|  | |  |  |  |  | 44 | 2.2 | | 92.4 |
|  | |  |  |  |  | 33 | 1.6 | | 94.0 |
|  | |  |  |  |  | 31 | 1.5 | | 95.5 |
|  | |  |  |  |  | 19 | 0.9 | | 96.4 |
|  | |  |  |  |  | 16 | 0.8 | | 97.2 |
|  | |  |  |  |  | 11 | 0.5 | | 97.7 |
|  | |  |  |  |  | 9 | 0.4 | | 98.1 |
|  | |  |  |  |  | 7 | 0.3 | | 98.4 |
|  | |  |  |  |  | 6 | 0.3 | | 98.7 |
|  | |  |  |  |  | 5 | 0.2 | | 98.9 |
|  | |  |  |  |  | 3 | 0.15 | | 99.1 |
|  | |  |  |  |  | 3 | 0.15 | | 99.2 |
|  | |  |  |  |  | 3 | 0.15 | | 99.4 |
|  | |  |  |  |  | 2 | 0.10 | | 99.5 |
|  | |  |  |  |  | 2 | 0.10 | | 99.6 |
|  | |  |  |  |  | 2 | 0.10 | | 99.7 |
|  | |  |  |  |  | 2 | 0.10 | | 99.8 |
|  | |  |  |  |  | 1 | 0.05 | | 99.9 |
|  | |  |  |  |  | 1 | 0.05 | | 99.9 |
|  | |  |  |  |  | 1 | 0.05 | | 100.0 |
|  | |  |  |  |  | 1 | 0.05 | | 100.0 |
|  | | | | | | | | | |
|  | Attended appointment | | | | | |  | Did not attend appointment | |

**PrEP: pre-exposure prophylaxis.**
