# Supplementary material for: High retention among key populations initiated on HIV pre‐exposure prophylaxis in Kigali City, Rwanda
Source: J Int AIDS Soc. 2024 Nov 20;27(11):e26392. doi: 10.1002/jia2.26392 (PMC11578929; doi:10.1002/jia2.26392)
Supplement: Supplementary file 2 — Table S1: Comparisons between individuals included in multivariable models and individuals excluded from models. Table S2: Retention at scheduled PrEP appointments among 2043 individuals initiating PrEP at 11 health centers in Kigali, Rwanda, 2019 2022. Table S3: Repeated measures analysis of PrEP retention among female sex workers utilizing generalized estimating equations, including data from 1‐, 3‐, 6‐, 9‐ and 12‐month visits (N = 1053). Table S4: Repeated measures analysis of PrEP retention among men who have sex with men utilizing generalized estimating equations, including data from 1‐, 3‐, 6‐, 9‐ and 12‐month visits (N = 558). Table S5: One‐ and twelve‐month PrEP retention at 11 health centers in Kigali, Rwanda. [file JIA2-27-e26392-s002.docx]

**SUPPLEMENTARY MATERIAL**

**Supplementary Table 1.** Comparisons between individuals included in multivariable models and individuals excluded from models.

|  | **FSW** | | | **MSM** | | | |
| --- | --- | --- | --- | --- | --- | --- | --- |
|  | Included | Not included | p | Included | Not included | | p |
| **Living Situation** | | | |  | | | |
| Live alone | 889 (84.4) | 71 (87.7) | 0.43 | 308 (55.2) | 37 (30.6) | | <0.001 |
| Live with others | 164 (15.6) | 10 (12.3) |  | 250 (44.8) | 84 (69.4) | |  |
| **Education level** | | | |  | | | |
| None | 119 (11.3) | 10 (11.4) | 0.99 | 12 (2.2) | | 2 (1.5) | 0.61 |
| Some Education (Primary or +) | 934 (88.7) | 78 (88.6) |  | 546 (97.8) | | 134 (98.5) |  |
| **Employment status** | | | |  | | | |
| Full-time | 57 (5.4) | 5 (6.1) | 0.80 | 83 (14.9) | | 15 (12.3) | 0.02 |
| Part-time | 335 (31.8) | 22 (26.8) |  | 207 (37.1) | | 48 (39.3) |  |
| Student | 19 (1.8) | 2 (2.4) |  | 37 (6.6) | | 18 (14.8) |  |
| Jobless | 642 (61.0) | 53 (64.6) |  | 231 (41.4) | | 41 (33.6) |  |
| **Condomless sex prior to initial visit** | | | |  | | | |
| Yes | 763 (72.5) | 160 (58.8) | <0.0001 | 396 (71.0) | | 43 (76.8) | 0.36 |
| No | 290 (27.5) | 112 (41.2) |  | 162 (29.0) | | 13 (23.2) |  |
| **Consider oneself to be at high risk of HIV** | | | |  | | | |
| Yes | 992 (94.2) | 244 (92.4) | 0.28 |  | |  |  |
| No | 61 (5.8) | 20 (7.6) |  |  | |  |  |
| **Aware of PrEP prior to PrEP enrollment** | | | |  | | | |
| Yes | 654 (62.1) | 176 (68.2) | 0.07 | 448 (80.3) | | 47 (83.9) | 0.51 |
| No | 399 (37.9) | 82 (31.8) |  | 110 (19.7) | | 9 (16.1) |  |
| **Self-reported diagnosis and treatment for STI in 12 months prior to PrEP enrollment** | | | | | | | |
| Yes | 267 (25.4) | 20 (24.1) | 0.80 | 213 (38.2) | | 94 (71.8) | <0.001 |
| No | 786 (74.6) | 63 (75.9) |  | 345 (61.8) | | 37 (28.2) |  |
|  | | | | | | | |
| OR: odds ratio; CI: confidence interval | | | | | | | |

**FSW: Female Sex Workers, MSM: Men who have Sex with Men, PrEP: pre-exposure prophylaxis, STI: sexual transmitted infections.**

**Supplementary Table 2.** Retention at scheduled PrEP appointments among 2043 individuals initiating PrEP at 11 health centers in Kigali, Rwanda, 2019 – 2022.

|  | **FSW (N=1343)** | **MSM (N=700)** |
| --- | --- | --- |
|  | **N (%)** | **N (%)** |
| **Initiated PrEP** | 1343 | 700 |
| **Retained at 1-month** | 1239 (92.2) | 674 (96.3) |
| **Retained at 3-month s** | 1220 (90.8) | 665 (95.0) |
| **Retained at 6-months** | 1168 (87.0) | 601 (85.9) |
| **Retained at 9-months** | 1054 (78.4) | 576 (82.3) |
| **Retained at 12-months** | 1032 (76.8) | 573 (81.9) |
|  |  |  |
| **PrEP: pre-exposure prophylaxis; FSW: female sex workers; MSM: men who have sex with men** | | |

**Supplementary Table 3.** Repeated measures analysis of PrEP retention among female sex workers utilizing generalized estimating equations, including data from 1-, 3-, 6-, 9- and 12-month visits (N=1053).

| **Variable** | **Univariate OR (95%) CI)** | **P-value** | **Multivariate aOR (95% CI)** | **P-value** |
| --- | --- | --- | --- | --- |
| **Living Situation** | | | | |
| Live alone (ref) |  | | | |
| Live with others | 0.86 (0.60, 1.24) | 0.43 | 0.71 (0.49, 1.02) | 0.06 |
|  | | | | |
| **Education level** | | | | |
| None (ref) |  | | | |
| Some Education (Primary or +) | 1.19 (0.78, 1.80) | 0.43 | 1.15 (0.75, 1.75) | 0.53 |
|  | | | | |
| **Employment status** | | | | |
| Full-time (ref) |  | | | |
| Part-time | 1.55 (0.83, 2.87) | 0.17 | 1.35 (0.72, 2.53) | 0.35 |
| Student | 1.38 (0.44, 4.30) | 0.58 | 2.14 (0.61, 7.58) | 0.24 |
| Jobless | 1.04 (0.57, 1.88) | 0.92 | 0.97 (0.53, 1.80) | 0.93 |
|  | | | | |
| **Condomless sex prior to initial visit** | | | | |
| Yes (ref) |  | | | |
| No | **0.76 (0.59, 0.97)** | **0.03** | 1.05 (0.77, 1.44) | 0.74 |
|  | | | | |
| **Consider oneself to be at high risk of HIV** | | | | |
| Yes (ref) |  | | | |
| No | **0.28 (0.19, 0.42)** | **<0.0001** | **0.20 (0.12, 0.32)** | **<0.0001** |
|  | | | | |
| **Aware of PrEP prior to PrEP enrollment** | | | | |
| Yes (ref) |  | | | |
| No | **1.38 (1.07, 1.77)** | **0.01** | 1.25 (0.91, 1.72) | 0.16 |
|  | | | | |
| **Self-reported diagnosis and treatment for STI in 12 months prior to PrEP enrollment** | | | | |
| Yes (ref) |  | | | |
| No | 1.02 (0.74, 1.40) | 0.91 | 0.87 (0.62, 1.22) | 0.41 |
|  | | | | |
| OR: odds ratio; CI: confidence interval | | | | |

**PrEP: pre-exposure prophylaxis, STI: sexual transmitted infections.**

**Supplementary Table 4.** Repeated measures analysis of PrEP retention among men who have sex with men utilizing generalized estimating equations, including data from 1-, 3-, 6-, 9- and 12-month visits (N=558).

| **Variable** | **Univariate OR (95% CI)** | **P-value** | **Multivariate aOR (95% CI)** | **P-value** |
| --- | --- | --- | --- | --- |
| **Living Situation** | | | | |
| Live alone (ref) |  | | | |
| Live with others | **0.42 (0.27, 0.65)** | **<0.001** | 1.02 (0.64, 1.63) | 0.94 |
|  |  |  |  |  |
| **Education level** | | | | |
| None (ref) |  | | | |
| Some Education (Primary or +) | 0.69 (0.07, 7.08) | 0.76 | 3.29 (0.72,14.96) | 0.12 |
|  |  |  |  |  |
| **Employment status** | | | | |
| Full-time (ref) |  | | | |
| Part-time | 0.69 (0.35, 1.33) | 0.26 | **0.29 (0.11, 0.76)** | **0.01** |
| Student | **0.32 (0.14, 0.75)** | **<0.01** | **0.12 (0.04, 0.37)** | **<0.001** |
| Jobless | **0.39 (0.20, 0.73)** | **<0.01** | **0.12 (0.05, 0.28)** | **<0.0001** |
|  |  |  |  |  |
| **Condomless sex since prior to initial visit** | | | | |
| Yes (ref) |  | | | |
| No | 2.02 (0.76, 5.37) | 0.16 | 2.42 (0.75, 7.86) | 0.14 |
|  |  |  |  |  |
| **Aware of PrEP prior to PrEP enrollment** | | | | |
| Yes (ref) |  | | | |
| No | **0.09 (0.06, 0.15)** | **<0.0001** | **0.15 (0.10, 0.23)** | **<0.0001** |
|  |  |  |  |  |
| **Self-reported diagnosis and treatment for STI in 12 months prior to PrEP enrollment** | | | | |
| Yes (ref) |  | | | |
| No | **2.74 (1.84, 4.07)** | **<0.0001** | 0.96 (0.60, 1.54) | 0.87 |
|  |  |  |  |  |
| OR: odds ratio; CI: confidence interval | | | | |

**PrEP: pre-exposure prophylaxis, STI: sexual transmitted infections.**

**Supplementary Table 5.** One- and twelve-month PrEP retention at 11 health centers in Kigali, Rwanda

| **Health Center** | **Number of patients included** | **Retained at 1 month** | **Retained at 12 months** |
| --- | --- | --- | --- |
| Bethsaida | 147 | 117 (79.6) | 52 (35.4) |
| Biryogo*^ | 292 | 289 (99.0) | 234 (80.1) |
| Busanza | 157 | 136 (86.6) | 55 (35.0) |
| Gahanga^ | 310 | 277 (89.4) | 260 (83.9) |
| Gatenga*^ | 355 | 351 (98.9) | 249 (98.3) |
| Gikondo | 138 | 125 (90.6) | 101 (73.2) |
| Kicukiro^ | 178 | 173 (97.2) | 169 (94.9) |
| Masaka | 148 | 138 (93.2) | 111 (75.0) |
| Nyarugunga | 129 | 129 (100.0) | 123 (95.4) |
| Remera* | 124 | 122 (98.4) | 120 (96.4) |
| We- Act for Hope | 65 | 56 (86.2) | 31 (47.7) |
|  |  |  |  |
| * considered a key population-friendly health center  ^ considered a larger (versus smaller) PrEP program  **PrEP: pre-exposure prophylaxis.** | | | |
